# Monitoring International Travelers Arriving in Hong Kong for Genomic Surveillance of SARS-CoV-2

**DOI:** 10.3201/eid2801.211804

**Published:** 2022-01

**Authors:** Haogao Gu, Samuel S.M. Cheng, Pavithra Krishnan, Daisy Y.M. Ng, Lydia D.J Chang, Gigi Y.Z. Liu, Sammi S.Y. Cheuk, Mani M.Y. Hui, Mathew C.Y. Fan, Jacob H.L. Wan, Leo H.K. Lau, Daniel K.W. Chu, Vijaykrishna Dhanasekaran, Malik Peiris, Leo L.M. Poon

**Affiliations:** University of Hong Kong School of Public Health, Hong Kong, China (H. Gu, S.S.M. Cheng, P. Krishnan, D.Y.M. Ng, L.D.J. Chang, G.Y.Z. Liu, S.S.Y. Cheuk, M.M.Y. Hui, M.C.Y. Fan, J.H.L. Wan, L.H.K. Lau, D.K.W. Chu, V. Dhanasekaran, M. Peiris, L.L.M. Poon);; HKU-Pasteur Research Pole, University of Hong Kong, Hong Kong (V. Dhanasekaran, M. Peiris, L.L.M. Poon);; Centre for Immunology and Infection, Hong Kong (M. Peiris, L.L.M. Poon)

**Keywords:** coronavirus disease, COVID-19, public health surveillance, respiratory infections, SARS-CoV-2, severe acute respiratory syndrome coronavirus 2, viruses, Hong Kong

## Abstract

We sequenced ≈50% of coronavirus disease cases imported to Hong Kong during March–July 2021 and identified 70 cases caused by Delta variants of severe acute respiratory syndrome coronavirus 2. The genomic diversity detected in Hong Kong was similar to global diversity, suggesting travel hubs can play a substantial role in surveillance.

Severe acute respiratory syndrome coronavirus 2 (SARS-CoV-2) lineage B.1.617 (*1*) and 3 of its sublineages, B.1.617.1 (Kappa), B.1.617.2 (Delta), and B.1.617.3, were first detected in India. The Delta variant started circulating widely in different continents beginning in late March 2021 (*2*,*3*). It was initially classified as a variant of interest in April 2021 and then reclassified as a variant of concern in May 2021.

Hong Kong adopted an elimination strategy to control coronavirus disease (COVID-19). A previous study reported the use of stringent measures (e.g., mandatory COVID-19 testing, travel restrictions) to detect and prevent SARS-CoV-2 importation by COVID-19–positive travelers (*4*), thereby reducing the risk of new SARS-CoV-2 introductions, and also showed that regional and international airports could be useful sentinel surveillance sites to monitor SARS-CoV-2 circulation. In this study, we tested the feasibility of using surveillance strategies similar to those used in that study to monitor sequence diversity of Delta variant SARS-CoV-2 among incoming travelers. Detection of B.1.617 variants at the end of March 2021 (*4*) prompted us to increase our sequencing efforts on imported COVID-19 cases. A total of 433 COVID-19 cases confirmed by reverse transcription PCR (RT-PCR) were imported during March 27–July 16, 2021; these cases accounted for 85.3% of all RT-PCR–confirmed COVID-19 cases in Hong Kong. We sequenced 49% (212) of those imported cases using next-generation sequencing technology (Appendix 1) and identified 42 Kappa and 70 Delta variant cases (Table). The same study reported that ≈80% of all imported COVID-19 cases in Hong Kong were asymptomatic at the time of RT-PCR testing (*4*). In this study, we observed a similar proportion (80.9%, N = 34) of asymptomatic Kappa variant cases but found that a significantly lower proportion, 52.8% (37/70), of Delta variant cases were asymptomatic (p<0.001 by χ^2^ test). This observation aligns with previous findings that the Delta variant virus can induce more severe clinical symptoms (*5*).

**Table:** Severe acute respiratory syndrome coronavirus 2 lineages identified from coronavirus disease cases imported to Hong Kong, March 27–July 16, 2021*

Date	Lineage	Total no. cases	Countries affected (no. cases)
Mar 27–31	B.1.1.7	6	Pakistan (2), Philippines (3), Turkey (1)
	P.3	5	Philippines (4), United States (1)
	B.1	1	Philippines (1)
	B.1.351	1	Philippines (1)
	B.1.466.2	1	Indonesia (1)
	B.1.526	1	United States (1)
	B.1.617.1	1	India (1)
	B.1.617.2	1	India (1)
Apr 1–30	B.1.617.1	41	India (41)
	B.1.1.7	38	Canada (1), France (2), India (9), Japan (1), Nepal (3), Pakistan (10), Philippines (10), Turkey (1), United States/Turkey (1)
	B.1.617.2	24	India (11), Nepal (13)
	B.1.351	16	Indonesia (1), Kenya (1), Philippines (14)
	B.1.466.2	7	Indonesia (7)
	B.1.470	2	Indonesia (2)
	C.36.3	2	Egypt (2)
	B.1	1	Philippines (1)
	B.1.1	1	Philippines (1)
	B.1.351.3	1	Bangladesh (1)
	B.1.36.18	1	Canada (1)
	B.1.441	1	Indonesia (1)
	B.1.456	1	Pakistan (1)
	P.3	1	Indonesia (1)
May 1–31	B.1.617.2	5	France (1), India (1), Nepal (3)
	B.1.441	2	Indonesia (2)
	B.1	1	Philippines (1)
	B.1.1.317	1	Russia (1)
	B.1.1.7	1	UAE (1)
	B.1.36	1	Pakistan (1)
	B.1.470	1	Indonesia (1)
Jun 1–30	B.1.617.2	35	Bangladesh (4), Indonesia (14), Namibia (1), Russia (1), United Arab Emirates (2), United Kingdom (13)
	B.1.1.7	2	Indonesia (2)
	B.1	1	France (1)
	B.1.351	1	Philippines (1)
	B.1.466.2	1	Indonesia (1)
	B.1.621	1	Colombia (1)
Jul 1–16	B.1.617.2	5	Cyprus (1), Ghana (1), Russia (3)

All Kappa variant cases were imported from India, where the Kappa variant predominantly circulated (*6*). In contrast, the Delta variant cases were imported from 11 countries in Asia, Europe, and Africa (Table). Delta variant cases within a specific country often clustered together temporally (Figure), mainly because additional travel bans to countries with high COVID-19 circulation during the study period were implemented, preventing introduction of more cases from these countries to Hong Kong. For example, because of the COVID-19 upsurge in India in early April 2021, all passenger flights from India were prohibited from landing in Hong Kong after April 19. Similar travel restrictions were also imposed on flights from Brazil, Indonesia, Nepal, Pakistan, Philippines, South Africa, and the United Kingdom at different times during the study period. These travel restrictions enabled us to capture viral sequence information from specific countries within limited identifiable time periods. Despite these restrictions, all 4 sublineages of B.1.617.2 (Delta I–IV) previously detected in other geographic locations (*7*) were detected in cases imported to Hong Kong (Appendix 1 Figure 1). Furthermore, we first detected these sublineages when they were in the early stages of global circulation (Appendix 1 Figure 2).

**Figure:**
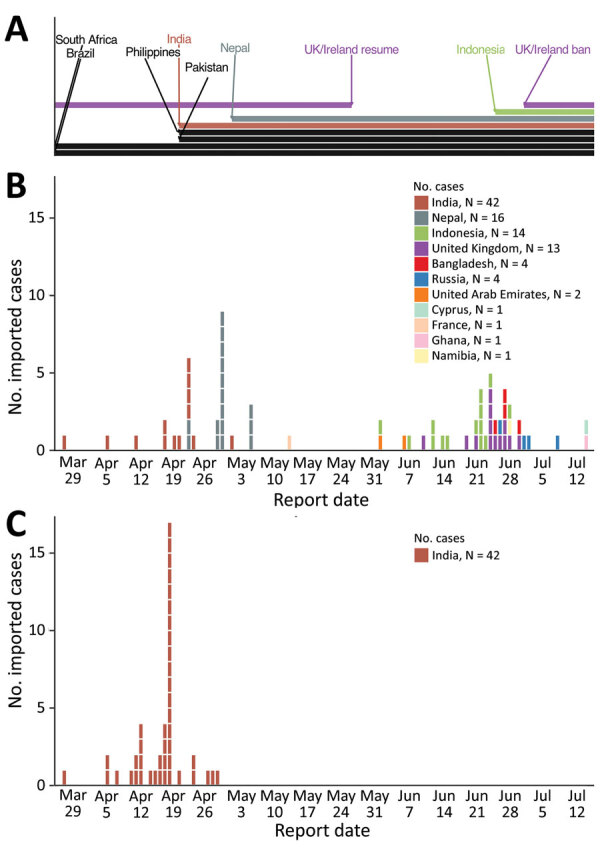
Imported Delta and Kappa variant severe acute respiratory syndrome coronavirus 2 infections, Hong Kong, March 27–July 16, 2021. A) Travel ban periods from each country to Hong Kong. B) Confirmed Delta variant cases, by country of origin. C) Confirmed Kappa variant cases; all were from India. All infections were confirmed by full-genome sequencing.

Although genomic sequencing has been used extensively to track SARS-CoV-2 transmission in specific geographic locations (*8*,*9*; H. Gu et al., unpub. data, https://doi.org/10.1101/2021.06.19.21259169), a global-level surveillance network using airports in different geographic locations might enable more feasible and cost-effective worldwide genomic surveillance. SARS-CoV-2 sequence information thus obtained, combined with relevant metadata, could strengthen current surveillance systems designed for other travel-related sources of illness and death (*10*). Specifically, we propose a multicenter surveillance network incorporating >1 travel hubs from each subcontinent. Sample selections would ideally be proportional to the number of confirmed imported cases by countries of origin and by time. Additional efforts should be made to study cases imported from countries with limited public sequencing information. Data required for sharing would at a minimum include genomic sequences, sample locations, and sampling dates. To avoid data de-anonymization, use of nonessential data (e.g., sex, age) could be restricted in or excluded from reports. The proposed surveillance network would take advantage of existing sequence-sharing platforms (e.g., GISAID, https://www.gisaid.org), but specific electronic tools and pipelines would need to be developed to enable timely, robust analyses. 

During the study period, we found only sporadic local COVID-19 cases in Hong Kong (N = 10). There were 2 independent local Delta variant cases in which the infection was acquired at the Hong Kong airport (Appendix 1 Figure 1). For regions using elimination strategies to control COVID-19, these findings suggest that airports can be high-risk settings for transmitting SARS-CoV-2 and introducing new variants. The findings also support use of stringent control measures and guidelines for protecting staff who work in airports. Overall, our results suggest that key travel hubs can effectively be used as valuable surveillance sites to monitor SARS-CoV-2 sequence diversity.

Virus sequences reported in this study are available from GISAID (https://www.gisaid.org; Appendix 2 Table 1). The data and analyzing scripts used in the study can be accessed in a GitHub repository (https://github.com/Leo-Poon-Lab/HK-Delta-variants).

Appendix 1 Additional information about monitoring incoming travelers arriving at Hong Kong for genomic surveillance of SARS-CoV-2.

Appendix 2 GISAID information for study of monitoring incoming travelers arriving at Hong Kong for genomic surveillance of SARS-CoV-2.
