# Supplementary material for: Monitoring International Travelers Arriving in Hong Kong for Genomic Surveillance of SARS-CoV-2
Source: Emerg Infect Dis. 2022 Jan;28(1):247–50. doi: 10.3201/eid2801.211804 (PMC8714226; doi:10.3201/eid2801.211804)
Supplement: Appendix 1 — Additional information about monitoring incoming travelers arriving at Hong Kong for genomic surveillance of SARS-CoV-2. [file 21-1804-Techapp-s1.pdf]

Article DOI: https://doi.org/10.3201/eid2801.211804

Monitoring International Travelers Arriving in Hong Kong for Genomic Surveillance of SARS-CoV-2

Appendix

Supplementary Methods

Sequencing

To obtain high quality sequence results, we only selected clinical samples with a cycle threshold value <30 for the next-generation sequencing study. RNA samples of interest were sent to a World Health Organization reference laboratory at the University of Hong Kong for full genome analyses (IRB number: UW 20–168). We deduced near–full-length genomes from all available samples using an Illumina (https://www.illumina.com) sequencing protocol previously described by us (*1*,*2*). Briefly, the virus genome was reverse transcribed with multiple gene-specific primers targeting different regions of the viral genome. The synthesized cDNA was then subjected to multiple overlapping 2 kb PCRs for full-genome amplification. PCR amplicons obtained from the same specimen were pooled and sequenced using Nova sequencing platform (Illumina). The sequencing library was prepared by Nextera XT (Illumina). Generated sequencing reads were mapped to a reference virus genome by BWA-MEM2 (https://github.com/lh3/bwa) (*3*), and genome consensus was generated by iVar with the PCR primer trimming protocol (minimum sequence depth >100 for NovaSeq and >10 for iSeq samples (*4*). Deduced consensus sequences with genome coverage <70% or failed to pass quality check of PANGOLIN (https://cov-lineages.org/resources/pangolin.html) were excluded from the downstream analyses. A total of 258 imported cases were sequenced in this study; 46 yielded unsatisfactory results and were excluded in the downstream analyses.

Epidemiologic Data

The metadata of the imported cases including case report date, case arrival date, country of importation are freely available in the Hong Kong governmental Web sites (*5*). To avoid de-anonymization of the genetic sequences, all the sequences submitted to GISAID (https://platform.gisaid.org) were named by in-house sample identifier rather than official

case number. The sex and age of the cases are not provided by default. The sample collection/report dates were only revealed in year-month format.

Phylogenetic Analysis

Maximum likelihood phylogenies were estimated using IQ-TREE (v.2.1.3) (*6*), employing the JC nucleotide substitution model with Wuhan-Hu-1 (GenBank: MN908947.3) as the outgroup. Dating of the Hong Kong tree and reconstruction of the ancestral node sequences were performed using Treetime v 0.8.1 (https://github.com/neherlab/treetime) (*7*). Lineages were classified using PANGOLIN (v.3.1.7) (pangoLEARN version 2021–07–09) (*8*). The global sequences were subsampled evenly (N = 3, per country per week, total N = 1,380) from all the high-quality Delta-variant sequences from GISAID (assessed 2021–06–28).

Data Availability

Virus sequences reported in this study are available from GISAID (Appendix Table 1).The data and analyzing scripts used in the study can be accessed in a GitHub repository (https://github.com/Leo-Poon-Lab/HK-Delta-variants).

Local Cases Linked to the Airport

Case-patient A was a ground staff person who escorts aircrews in the airport. Epidemiologic investigations revealed that patient A had brief encounters with a COVID-19 cluster imported from Indonesia in the airport (*9*), and our genomic analyses of these cases confirmed the findings (Figure, case A, cluster 32). Case-patient B was a porter at the airport. Epidemiologic investigations revealed that patient B might have acquired the infection during his stay in a Russian cargo aircraft which briefly stayed in Hong Kong. Our sequence analyses also confirmed that the viral sequence from patient B was genetically most similar to our cases imported from Russia (Figure, clusters 27 and 28).

References

1. Choi EM, Chu DKW, Cheng PKC, Tsang DNC, Peiris M, Bausch DG, et al. In-flight transmission of SARS-CoV-2. Emerg Infect Dis. 2020;26:2713–6. PubMed https://doi.org/10.3201/eid2611.203254

2. Sit THC, Brackman CJ, Ip SM, Tam KWS, Law PYT, To EMW, et al. Infection of dogs with SARS-CoV-2. Nature. 2020;586:776–8. PubMed https://doi.org/10.1038/s41586-020-2334-5

3. Vasimuddin M, Misra S, Li H, Aluru S. Efficient architecture-aware acceleration of BWA-MEM for multicore systems. 2019 IEEE International Parallel and Distributed Processing

Symposium (IPDPS); 2019 May 20–24; Rio de Janeiro, Brazil. Piscataway, NJ, USA: IEEE; 2019. p. 314–24.

4. Grubaugh ND, Gangavarapu K, Quick J, Matteson NL, De Jesus JG, Main BJ, et al. An amplicon-based sequencing framework for accurately measuring intrahost virus diversity using PrimalSeq and iVar. Genome Biol. 2019;20:8. PubMed https://doi.org/10.1186/s13059-018-1618-7

5. Hong Kong Government. Data in coronavirus disease (COVID-19). [cited 2021 Aug 19] https://data.gov.hk/en-data/dataset/hk-dh-chpsebcddr-novel-infectious-agent

6. Minh BQ, Schmidt HA, Chernomor O, Schrempf D, Woodhams MD, von Haeseler A, et al. IQ-TREE 2: new models and efficient methods for phylogenetic inference in the genomic era. [Erratum in Mol Biol Evol. 2020;37:2461.]. Mol Biol Evol. 2020;37:1530–4. PubMed https://doi.org/10.1093/molbev/msaa015

7. Sagulenko P, Puller V, Neher RA. TreeTime: Maximum-likelihood phylodynamic analysis. Virus Evol. 2018;4:vex042. PubMed https://doi.org/10.1093/ve/vex042

8. O'Toole A, Scher E, Underwood A, Jackson B, Hill V, McCrone JT, et al. Assignment of epidemiological lineages in an emerging pandemic using the pangolin tool. Virus Evol. 2021;7:veab064.

9. Hong Kong Government. CHP investigates one additional confirmed case of COVID-19 and amends case classification. [cited 2021 Aug 19] https://www.info.gov.hk/gia/general/202106/29/P2021062900391.htm

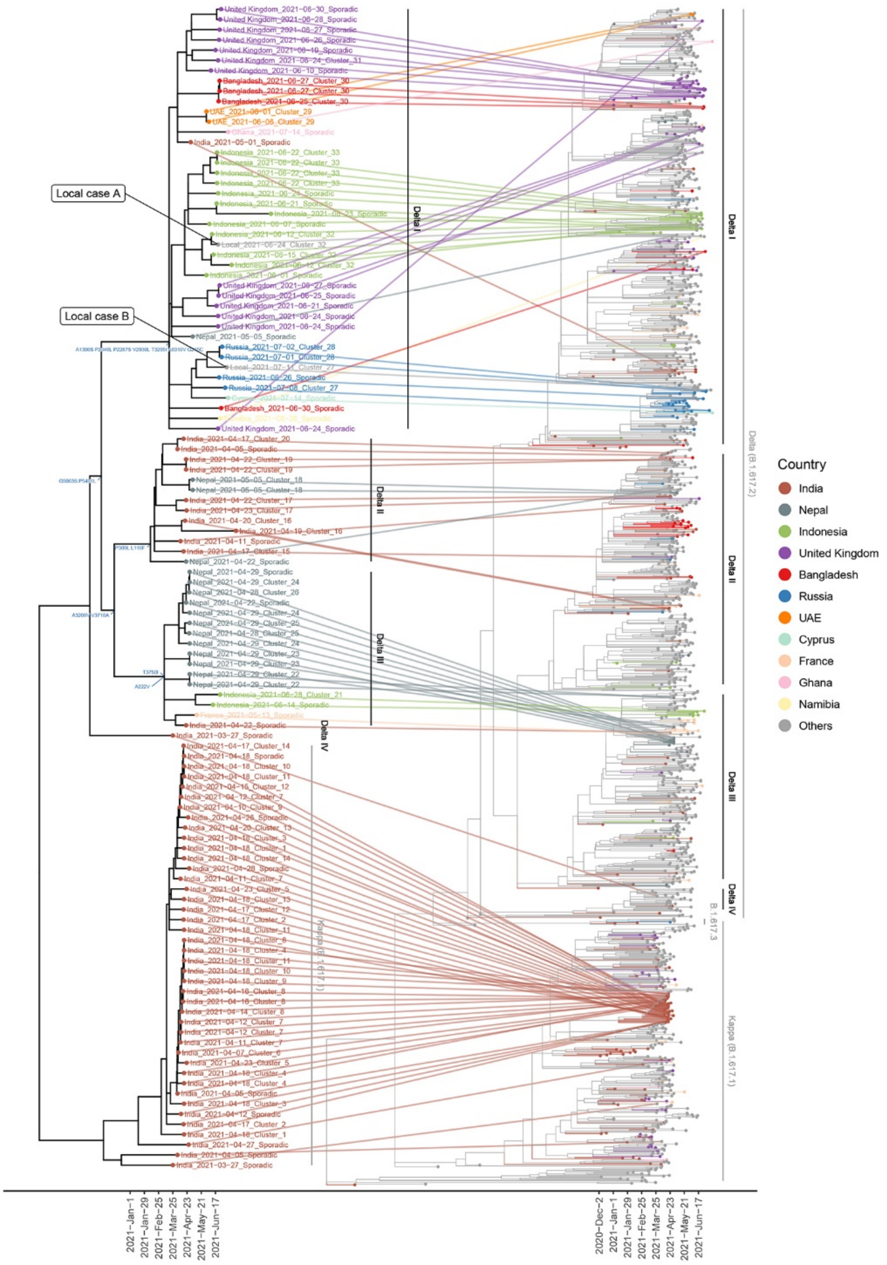

Appendix Figure 1. Phylogeny of SARS-CoV-2 B.1.617 variants identified in Hong Kong (left) and globally (right). The tips in the trees are colored according to the countries of origins. The global sequences were subsampled evenly (N = 3 per country per week, total N = 1,380) from all the high-quality Delta-variant sequences from GISAID (assessed 28 Jun 2021). Mutations defining the Delta sublineages were labeled at the ancestral nodes (*8*). Cases imported to Hong Kong that were epidemiologically linked are labeled with the same cluster number. Maximum likelihood phylogenies were estimated using IQ-TREE (v.2.1.3) (https://www.iqtree.org), employing the JC nucleotide substitution model with Wuhan-Hu-1 (GenBank accession no. MN908947.3) as the outgroup. Dating of the trees and reconstruction of the ancestral node sequences were performed using Treetime (v0.8.1) (https://treetime.readthedocs.io/en/latest). Lineages were classified using PANGOLIN (v3.1.7) (https://cov-lineages.org/resources/pangolin.html).

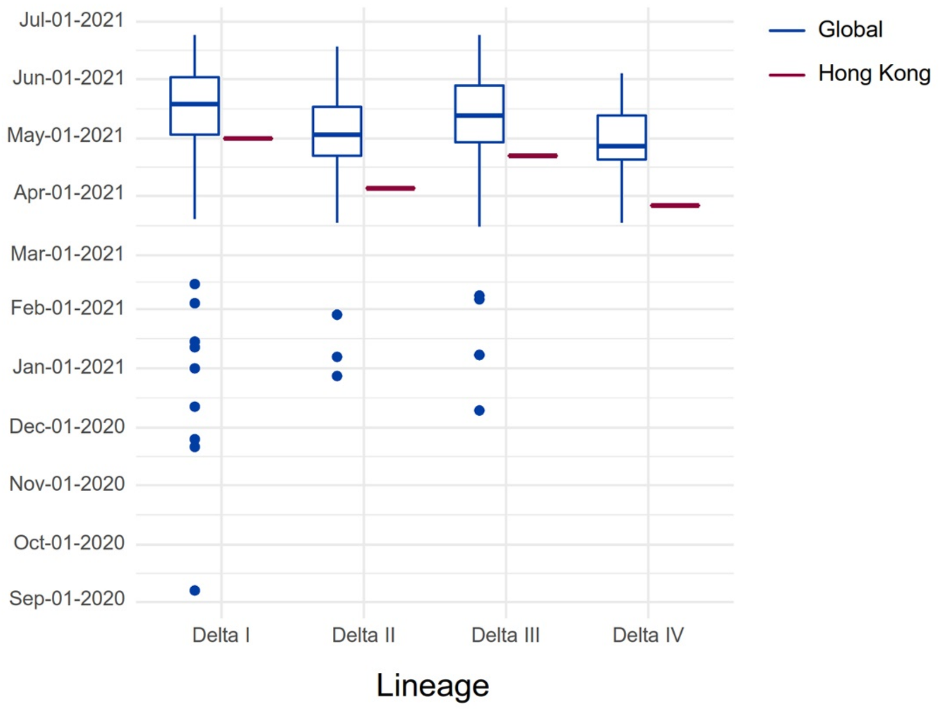

Appendix Figure 2. Detection dates of Delta variant sequences reported in the GISAID database (https://platform.gisaid.org). Distribution of dates of global subsampled data shown in box plots for different Delta sublineages. Dates represent outliers, minimum, 1st quartile, median, 3rd quartile, and maximum of each sublineage. Red line indicates detection date in Hong Kong for each sublineage.
